# Relative expression of TAp73 and ΔNp73 isoforms

**DOI:** 10.18632/aging.100441

**Published:** 2012-03-04

**Authors:** Franco Conforti, Ai Li Yang, Massimiliano Agostini, Alessandro Rufini, Paola Tucci, Maria Victoria Nicklison-Chirou, Francesca Grespi, Tania Velletri, Richard A. Knight, Gerry Melino, Berna S. Sayan

**Affiliations:** ^1^ University of Southampton, Faculty of Medicine, Cancer Sciences Unit, Somers Cancer Research Building, Southampton, SO16 6YD, UK; ^2^ Medical Research Council, Toxicology Unit, Hodgkin Building, Leicester University, Leicester LE1 9HN, UK; ^3^ Biochemistry Laboratory, IDI-IRCCS, and Department of Experimental Medicine and Biochemical Sciences, University of Rome “Tor Vergata”, 00133 Rome, Italy

**Keywords:** p73, alternative splicing, expression, cancer

## Abstract

The transcription factor p73 belongs to the p53 family of tumour suppressors and similar to other family members, transcribed as different isoforms with opposing pro- and anti-apoptotic functions. Unlike p53, p73 mutations are extremely rare in cancers. Instead, the pro-apoptotic activities of transcriptionally active p73 isoforms are commonly inhibited by over-expression of the dominant negative p73 isoforms. Therefore the relative ratio of different p73 isoforms is critical for the cellular response to a chemotherapeutic agent. Here, we analysed the expression of N-terminal p73 isoforms in cell lines and mouse tissues. Our data showed that the transcriptionally competent TAp73 isoform is abundantly expressed in cancer cell lines compared to the dominant negative ΔNp73 isoform. Interestingly, we detected higher levels of ΔNp73 in some mouse tissues, suggesting that ΔNp73 may have a physiological role in these tissues.

The Trp73 gene belongs to the p53 family of transcription factors and, like the other members, is transcribed into different isoforms [1-4]. TP73 gene contains two promoters, encoding the transcriptional domain-containing (TAp73) and the amino deleted (ΔNp73) isoforms [5, 6]. Furthermore alternative splicing at the 3'-end (to generate α, β, γ, etc isoforms) and 5'-end (to generate Δ2, Δ3 and Δ2-3 isoforms) results in generation of at least 14 different transcripts, with different abilities to promote or repress apoptosis [7, 8]. DNA damaging agents induce TAp73 and activate p73-dependent gene expression program to promote cell cycle arrest and apoptosis. Interestingly, ΔNp73 can counteract these activities, either by directly binding and inhibiting transcription or by competing for DNA binding sites [9-13]. This suggests that the overall activity of p73 stems from the relative expression level of each isoform, rather than the independent activities of single isoforms. The relative expression level is maintained both at transcriptional level and post-translational level. While p73 levels are regulated via acetylation, phosphorylation, interaction with PML, caspase cleavage and degradation by the ubiquitin ligase ITCH [14-19], ΔNp73 isoforms are selectively degraded by the E3 ubiquitin ligase PIR2 or via the antizyme pathway [20, 21]. Although experimental evidence and epidemiological studies point to a role of p73 in cancer [22-24], clear, unbiased data are only very recent [25, 26]. Direct mutations of p73 in tumours are rare, but several studies have revealed a clear tendency towards upregulation of ΔNp73 isoforms and methylation dependent silencing of TAp73, resulting in an imbalance of TA/ΔN ratio. Furthermore, recently developed isoform specific knockout mice have shown that depletion of TAp73 predisposes to cancer, while absence of ΔNp73 impairs tumour growth in transplant assays [25, 26]. For these reasons, we sought to investigate the expression of p73 N-terminal isoforms in a panel of cell lines and mouse tissues. First, we used N-terminal selective primers to analyse the expression of TAp73 and ΔNp73 by qPCR in several cancer cell lines (Figure 1A). Surprisingly, we found consistently higher expression of TAp73 isoforms in the selected cell lines, with the exception of the T-cell leukemia Jurkat cells, where TAp73 and ΔNp73 wereequally expressed. It is noteworthy that in some cell lines like MCF7, COS7 and SW480 we found an impressive imbalance between the two isoforms with up to 100 fold higher TAp73 levels. Next, to validate qPCR data, we investigated protein expression in some of the previous cell lines. To this aim, we used a previously described pan-p73 antibody [27] and utilized p73 siRNA and overexpression of p73 isoforms as controls. Importantly, protein expression levels paralleled mRNA data. Indeed, TAp73α was the most abundant endogenous isoform detected in cells. As shown in Fig 1B/C/D, in most of the cell lines, we detected a 75kDa band that co-migrated with TAp73α positive control and was efficiently silenced by siRNA. Moreover, etoposide treatment in H1299 cell line led to the up-regulation of the 75kDa band (Figure1C), further substantiating efficient detection of TAp73 by the aforementioned antibody. In addition, we analysed p73 N-terminal isoforms expression in mouse tissues. Interestingly, analysis of TA/ΔN ratio, confirmed higher TAp73 expression in-vivo in many organs (Figure 1E). In fact, higher relative levels (>10 fold) of TAp73 mRNA were found in spleen, fat, kidney and bladder, while intermediate levels (<10 fold) were detected in liver, lung and gut. On the other hand, ΔNp73 was the main isoform detected in uterus, salivary gland and tongue. Finally, comparable levels of TA and ΔNp73 mRNAs were expressed in skin, brain, colon and ovary. In summary, we carried out an extensive study of p73 N-terminal isoforms expression both in human cancer cell lines and mouse tissues. Surprisingly, our data showed that transcriptionally competent TA isoforms were detectable at higher levels in most of the cell lines and tissues that were analysed. This is counterintuitive considering the current knowledge on p73, especially in cancer samples, as one would expect increased ΔNp73 expression to be a hallmark of cancer. Also this raises the question about the role of TAp73 in these cells and how its anti-proliferative functions are dealt with. Finally, data on N-terminal p73 isoform expression in primary tumours would be highly beneficial, as this would avoid any bias from prolonged in vitro culture.

**Figure 1:**
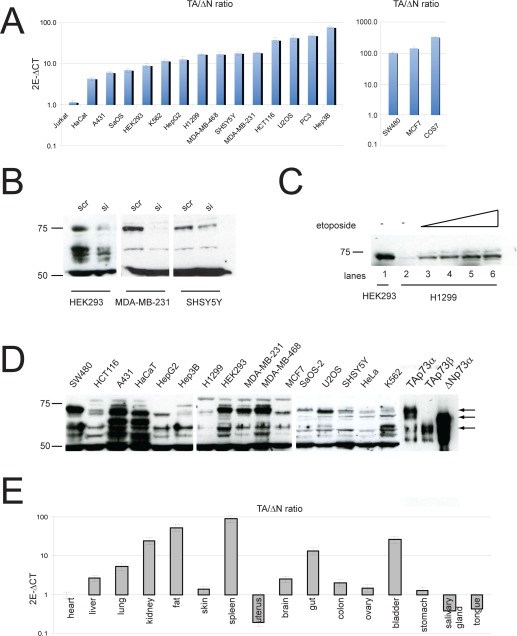
Expression of p73 isoforms (**A**) Total RNA was isolated from different cell lines as described before [5] and TAp73 and ΔNp73 expression were evaluated by real-time PCR with the TAp73 and ΔNp73 specific primers. (**B**-**C**) Validation of p73 antibody using either specific siRNA against p73 or induction of its expression by etoposide. Endogenous p73 was silenced in indicated cell lines and 50 ug protein was used to detect p73. Specificity of the antibody was also verified by detecting up-regulated TAp73 in H1299 cells following treatment with 20-50 uM etoposide for 24 h. (**D**) Western blot analysis of p73 isoforms in different cell lines. 50 ug protein was used to detect endogenous p73 protein. (**E**) Total RNA was isolated from different tissues and TAp73 and ΔNp73 expression were evaluated by real-time PCR as in panel **A**.

## Abbreviations

TAp73: transcriptional domain–containing active p73
*Δ*Np73: amino deleted p73
